# From steroids to aqueous supramolecular chemistry: an autobiographical career review

**DOI:** 10.3762/bjoc.12.69

**Published:** 2016-04-12

**Authors:** Bruce C Gibb

**Affiliations:** 1 Department of Chemistry, Tulane University, New Orleans, LA 70118, USA

**Keywords:** supramolecular chemistry

## Abstract

The focus of my group’s research is aqueous supramolecular chemistry; we try to understand how chemical entities interact with water and consequently how they interact with each other. This personal history recounts my career experiences that led to his involvement with this fascinating area of science.

## Review

**Scheme 1:**
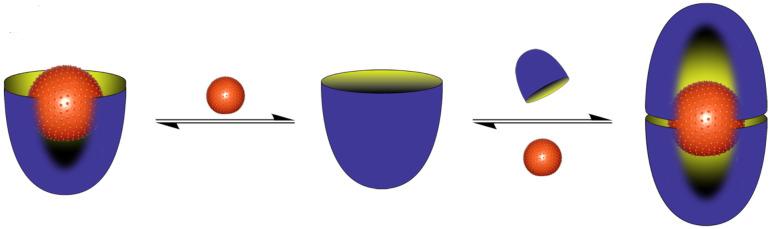
The formation of a 1:1 complex and a 2:1 supramolecular nano-capsule complex from bowl-shaped “cavitand” molecules.

I recall a conversation with J. Fraser Stoddart some years ago that now seems pertinent to this review. He asked a simple question, “what did your parents do for a living?” For the record, my father and dear departed mother worked together in their own watch-makers and jewelers. My father built and repaired timepieces, whilst my mother worked on the jewelry side of things. In contrast, my interests center round something more mundane, but devilishly more complicated: water. More specifically, my research group is interested in the interactions between water and species dissolved in it. In short: aqueous supramolecular chemistry. We investigate supramolecular processes in water in order to identify new and interesting phenomenons, and to contribute to Science’s understanding of the properties of aqueous solutions. As discussed below, the latter can be bifurcated into two general topics: the hydrophobic effect and the Hofmeister effect. However, specific molecular-level understandings of these phenomena are far from complete. We’ll return to this in due course, but a summary of a large part of our research – the formation of 1:1 complexes and 2:1 capsular complexes in aqueous solution – is shown in Scheme 1 [1].

**Scheme 2:**
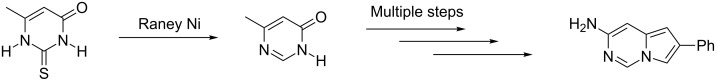
Abbreviated synthesis of 7-amino-2-phenyl-6-azaindolizine.

Why did Fraser Stoddart care about my parent’s occupation? The premise of the question, as Fraser said after I responded, was that he thought that supramolecular chemists had an artistic, visual-spatial bent. That’s certainly true in my case, when not delving into research my hobbies are photography and cooking; the former is very visual, the product of the latter is too, and both are artistic in their own right. Indeed, art is in my family; my father didn’t only enjoy the artistry of building and repairing watches and clocks, but he is also a keen watercolor artist. Moreover, my (elder) brother is a trained architect. It was my artistic side that initially drove me to consider going to university and studying architecture, but my brother beat me to designing buildings and urban landscapes. So a combination of my ego and my scientific persona drove me ultimately to the physical sciences; I attended Robert Gordon’s Institute of Technology in Aberdeen (Scotland) to study a degree in Physical Sciences, and in my third year I opted to major in Chemistry. If I wasn’t going to be designing buildings, I’d design molecules instead! The alternatives of physics or biochemistry also seemed like fun, but I was attracted to chemistry by the intrinsic beauty of organic chemistry. There is an ineffable artistry to a reaction scheme that, combined with the chess-like options of organic synthesis that have been built up over the centuries, can be as beautiful as it can be beguiling. And so, in my honors year it was time to work on a research project under the guidance of one of the graduate students in the department. Naturally, I opted for an organic project: The synthesis and reactivity of 7-amino-2-phenyl-6-azaindolizine (Scheme 2). Fortuitously, this project was to be guided “on the ground” by Derek McHattie, a third year Ph.D. student possessing a keen mind and a healthy disdain for conformity. As is often the case with undergraduate research, The Boss, in my case Dr. Martin Fraser, was “up at 35,000 feet” and was a lot less accessible than Derek. And so it was Derek, whom I’m fortunate enough to still consider a good friend, who really introduced me to organic chemistry research. Derek sent me to work on the first step, the unoptimized desulfurization of 2-thio-6-methyluracil using Raney-Nickel to form 6-methyl-4-hydroxypyrimidine (Scheme 2). It turned out I was a wiz at this organic synthesis lark (as Derek liked to say). I took the yield from 73% to quantitative by doing what organic chemists do, changing one parameter at a time whilst keeping an eye on the yield and the practicality of the reaction and workup. And so my first foray into organic synthesis was a success; I had gone mano-a-mano with Mother Nature and won. I was hooked! Of course, Nature was to put me in my place on plenty of other occasions; she truly does guard her secrets well. But I had the bug, and loved the never-ending competition. Those universal constants and physical laws are totally reliable and allow you to know your opponent to an acceptable (but far from complete!) degree. Picking a battle with Mother Nature is just a sheer delight.

**Figure 1:**
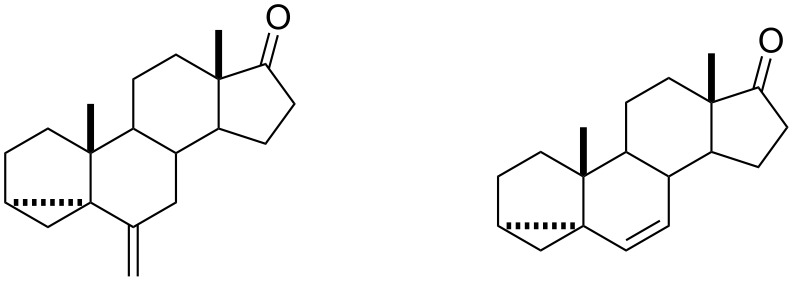
My two favorite compounds for my Ph.D. dissertation, “The Synthesis and Structural Examination of 3α,5-cyclo-5α-androstane Steroids”.

Subsequently I went on to study my Ph.D. degree. I opted for the same university, but switched to the Pharmacy Department to work on the synthesis of steroids under the tutelage of Dr. Philip J. Cox and Dr. Steve M. MacManus. Phil, an X-ray crystallographer, was my director of studies and he needed new steroids synthesized and structurally characterized [2–3]. So he brought on-board Steve – a natural products chemist – to guide me through the synthesis of the different targets. The overall goal of my work was the development of new steroidal contraceptives, and the key structural theme of the different androstane targets was the presence of a cyclopropane moiety in the A-ring. Figure 1 shows two of my favorite molecules synthesized in my work.

What fascinated me about these molecules – and I now realize that this was a sign of my physical-organic chemistry leanings – was the spectroscopic difference between these molecules and how these compared to analogous steroids whereby the cyclopropane group in the A-ring is replaced with a conjugating C=C double bond. To cut a long story short, vinyl cyclopropanes are conjugated, albeit to a lesser degree than dienes, and NMR, IR and UV–vis analysis showed up some fascinating differences between these steroids that could be related to their structures derived from molecular mechanics.

**Scheme 3:**
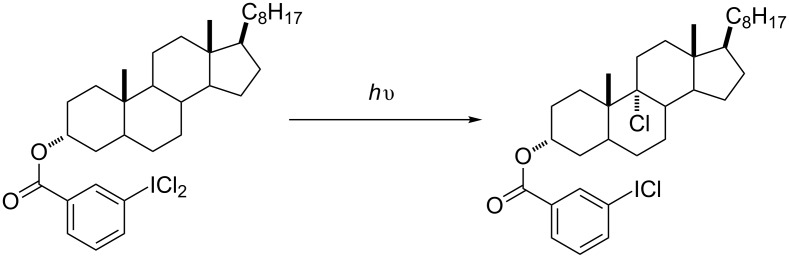
An inspiring chlorination from the group of Ronald Breslow.

This topic was not however the greatest, lasting memory from my Ph.D. studies. Neither was manually dredging the Chemical Abstracts for each literature search (if only the students of today could understand!). No, my greatest, lasting memory from my years as a Ph.D. student was an old paper I found from the labs of Ronald Breslow [4]. In particular, what caught my eye was the conversion of 3α-cholestanyl *m*-iodobenzoate dichloride to the corresponding 9α-chlorosteroid via internal hydrogen abstraction (Scheme 3).

It was not that I was trying to make 9-halogeneated derivatives, or indeed do anything remotely analogous to what Breslow was accomplishing. It was simply that having worked with steroids for a couple of years I appreciated that they really are just a, “slab of hydrocarbon”. Hence this example of selective, remote functionalization of the steroid framework was captivating. This captivation was itself pivotal to my career path, and it is a constant reminder to me (and hopefully young practitioners I’ve told this story to) how important it is to keep one’s eye on the literature at all times. Not by having an unknown algorithm carry out an automated keyword search each week, but by actually sitting down with a cup of your favorite drink and perusing individual journal issues. The simple fact is that you can’t catch important, interesting research peripheral to your current studies with SciFinder. Life is so much more than either your brain or an algorithm, and if you expose yourself to other chemistries going on around you might be very surprised what pops up.

In my case, the Breslow paper pointed me towards the cytochrome P450 family of enzymes, or more specifically, the subset of this class of enzymes that play an important role in steroidogenesis. How does the body synthesize all those steroid hormones? How does Nature carry out remote functionalization on these slabs of hydrocarbon? The answer of course involves a heme-cofactor and an exquisitely shaped hydrophobic pocket/active site. Ronald Breslow was asking similar questions and addressing it with cyclodextrin derivatives as enzyme mimics, but as I neared the end of my Ph.D. degree I confess I knew nothing of this facet of supramolecular chemistry.

**Scheme 4:**
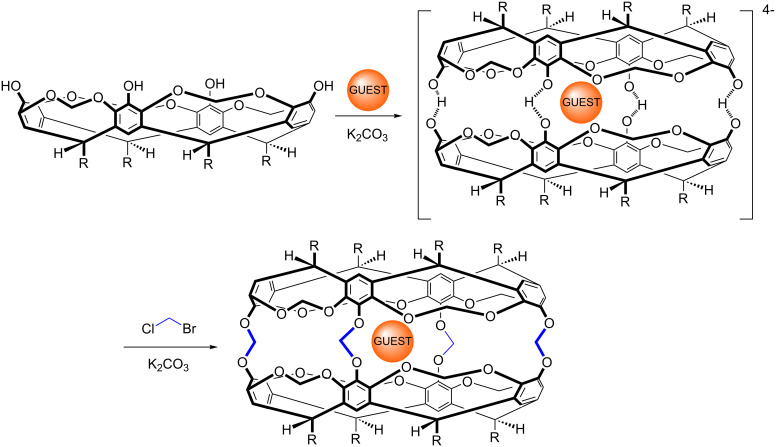
The carceplex reaction.

In 1991, as lab work started to be replaced by thesis writing I found myself with two concerns: 1) was I going to regret pioneering thesis writing at my department using Wordstar 5.0? 2) What was I going to do next? On the first topic, I think it was ultimately a good idea not to write my thesis by hand and then get it typed up, but I will never forget all the (physical) cutting and pasting of printed Chemdraw 2.0 images into gaps in my thesis text; what a drag! As for the more important task of the next step in my career, I was not happy with the sort of job interviews I was getting in the UK, and as a result, I decided to upend everything and move out to Vancouver, British Columbia (BC). Now this was not entirely on a whim, 18 months earlier my ultimately to be wife, Corinne, had moved there to carry out polymer research at the University of British Columbia (UBC) with Donald Brooks, so I figured a few months exploring options in the west coast of Canada would be at least fun and potentially fruitful. Upon arriving I gathered up all the department brochures I could get my hands on (yes, there was no internet) and quickly discovered a whole range of potential labs to work in. There were many programs of research at UBC, Simon Fraser University, and the University of Victoria that appealed to me, but of all the people I talked to, the work of John Sherman at UBC appealed the most. John had carried out his Ph.D. with Don Cram at UCLA right at the (Nobel Prize winning) pinnacle of Don’s career, and like so many people in the group at the time had worked with cavitands. In fact, John was – to cut a long story short – instrumental to Don’s publications on the synthesis and properties of soluble carceplexes formed by the dimerization and covalent linking of tetrol-cavitands (Scheme 4). When I first arrived on the scene one of John’s projects was to understand this templated reaction. I had discovered supramolecular chemistry! Alas, this very project was being driven by Bob Chapman; an outstanding chemist who’s now a Natural Health Products Program Leader at the National Research Council in Canada [5–7].

So the good news was that I had “discovered” molecular concavity and host–guest chemistry, research that I instantly related to the action of the Cytochrome P-450s and other enzymes. Unfortunately however, there was the small problem of financial support. From John’s perspective I had literally appeared out of the blue, so as there was no possibility of support I simply volunteered to take a gratis appointment. So I got to work; sustained by Corinne’s stipend that – thanks to my cooking skills – fed the two of us much more successfully than did Corinne’s cooking for one (pasta with fresh air anyone?).

**Figure 2:**
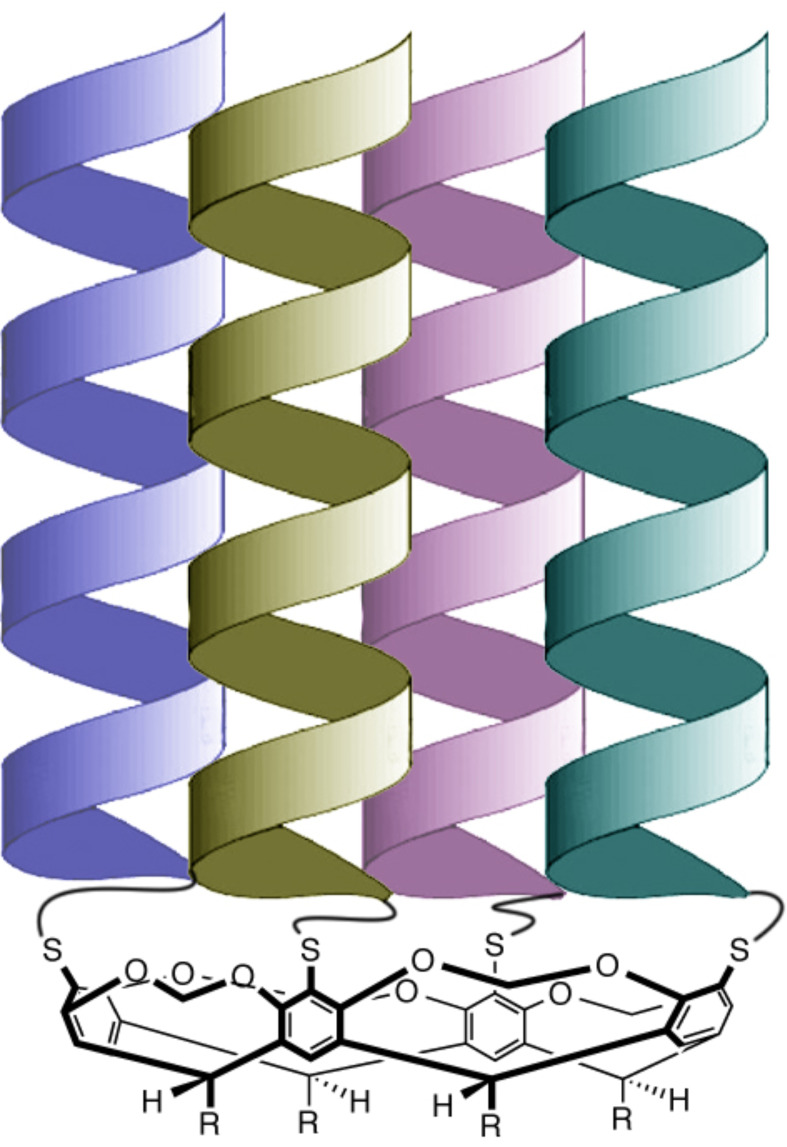
Schematic of a cavitein.

I had three related projects in John’s labs: one was to see if noble gases such as xenon could template the above reaction (short answer, not in my hands); one was to synthesize new cavitands with functionality at their feet (the R groups in Scheme 4) [8]; and the third was to devise a way in which four α-helical peptides could be coupled to the rim of a cavitand scaffold to form mimics of four-helix bundles or caviteins (Figure 2) [9–10]. These latter two projects worked well, although I ended up moving on before the cavitein work could be brought to full fruition; it was more a case of me ”clearing the brush” and setting things up for new graduate student Adam Meso to really start to bring out the best in the project [11]. But I’m jumping ahead of myself here. After a few months with John, and an unsuccessful RSC post-doc fellowship application to try and make my position more concrete, things improved considerably when John managed to find a way to support me. Now I was actually being paid to do what I wanted to do. I still don’t know where that money came from, but it was pivotal. Thanks John!

I made lots of friends and memories in beautiful BC, but career wise I recall two moments that had the greatest effect on my career. The first was when another faculty member whom I had initially spoken with whilst looking for a position, turned up in John’s lab to try to hire me away. I guess John had mentioned to his colleague that I was able to synthesize molecules and I had therefore suddenly become an attractive prospect. I turned my suitor down; he was a big-time player, but it was John who had shown me faith. That stated, the offer was a nice confidence-booster. Here I was at a department that was – at least in terms of number of researchers – literally fifty times larger than where I had completed my Ph.D.; but I was apparently still quite good at this “synthesis lark.” That calibration help me no end. The second BC-moment was a career chat with John. It was a simple conversation about what I was to do next, but it suddenly made me realize the obvious: that there was the option of staying in North America to continue my career. And so that is what I opted for; in large part because the prospect was so exciting, but also because having completed my studies in Scotland at a small school I felt a career in the UK would be hampered by the country’s predilection to put too much stock in pedigree. Looking into my options, John and I narrowed things down to either a post-doc with Donald Cram at UCLA, or a position at NYU with James Canary. The possibility of working with Don was provocative, but this was right at the end of his career and in all likelihood I would probably end up making carceplexes, and after working with John I wanted something new. And so I opted to swap the wilds of BC for the wilds of New York City. After a wonderfully exciting road trip across the continent, Corinne, and I arrived in Manhattan on a beautiful Sunday evening in October. The images of Bleecker Street and Greenwich Village life that evening will always be with me.

**Figure 3:**
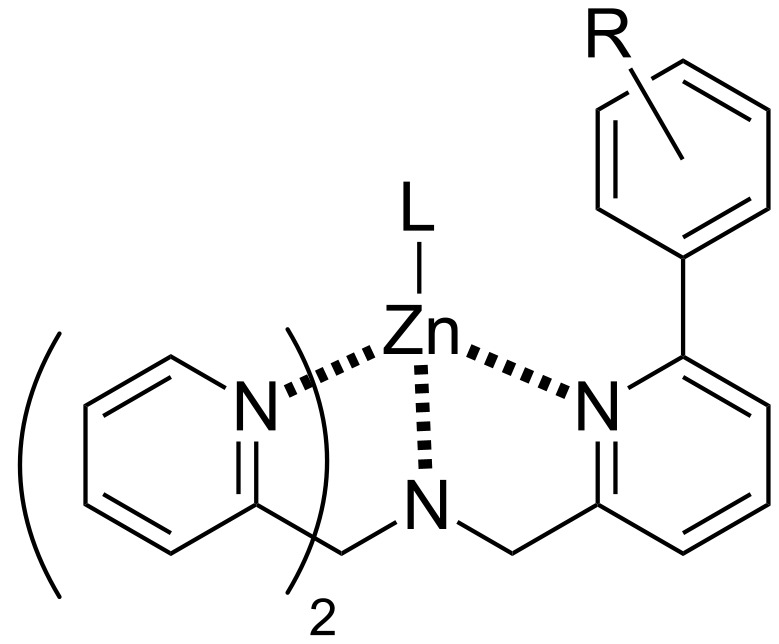
General structure of zinc-TPA complexes.

Living in Manhattan and working with Jim proved to be a real eye-opener. What fun! Jim and I turned out to be almost exact opposites; in a good way. My project was to synthesize some tris(2-pyridylmethyl)amine (TPA)-based ligands and study their zinc complexes (Figure 3) as mimics of the enzyme Carbonic Anhydrase. The ligands for these complexes are notoriously difficult to work with chromatographically, but I managed to synthesize a few derivatives that not only replicated the zinc binding site, but also some of the functionality that ‘dangles’ above the zinc-bound water in the crystal structure of the enzyme. In addition to synthesis, the project also involved a good deal of potentiometry, and this began to teach me that synthesizing enzyme mimics was a difficult challenge of balancing the need to create functionality for the active site, whilst maintaining suitable water solubility. Whilst I was busy doing all of this with Jim, Corinne was working in the labs of Neville Kallenbach, then Chair of the Chemistry Department. Corinne and Neville got on like a house on fire, and so I got to know Neville very well.

Jim also had a big influence on my career, and I can recall two key moments where his wisdom helped point me in the right direction. The first came as we wrote a review on metallo-enzyme mimics [12]. Near the end of the whole process I mentioned to Jim that it was kind of disappointing that when synthesizing an enzyme mimic nobody had really, truly integrated a hydrophobic pocket with the catalytic machinery required for whichever catalytic conversion was targeted. People such as Breslow had coupled these two facets of a shape-selective catalyst together, but no one had integrated these two aspects of an enzyme to the level Nature does. Arch critic that I am, I turned on my own research; “how can we ever expect to get the zinc-bound water in our complexes to be the strong acid it is in Carbonic Anhydrase if we’re not fully taking into account context?” Jim gave me his bemused/slightly-exasperated/are you kidding me kind of look – the one that can put anybody in their place – and said, “why don’t you go off and synthesize your own synthetic hydrophobic pocket and then you can add all the catalytic machinery you want.” Twenty years later, all I can say is that I’m still trying. Stay tuned Jim!

The other key conversation I had with Jim was very much career-related. I was leaning towards an academic job, but what sealed it for me was when Jim said words to the effect of, “Do you want to be a cog in a wheel, or do you want to be your own boss and take your own independent thoughts and turn them into something?” That kindled the independent streak within, and I thought of my mother and father and all my ancestors that had had their own businesses. Why not do the same in an academic setting and see what comes forth?

And so I applied for every academic job that came up that year: 54 there were, 2 in Canada, 2 in the UK, and 50 in the US. Considering my pedigree, some of the institutions were quite frankly out of my depth. In fact, to be honest, all of them arguably were. I just didn’t have the CV bulging with big important papers backed up by heavy-hitters in the field. Still, you’ve got to try don’t you? To cut a long story short, after many late nights of writing three proposals – with the invaluable help of Corinne and our good friend Niels Van der Veen running around the library digging up papers – everything cerebral was ready. Then I turned to our neighbor Julie Kaplan – the events and outreach guru at NYU Chemistry – to give myself and my stack of applications that certain *je ne sais quoi*. After spending all I could on everything from a respectable outfit for me to posh paper for my CV, I was ready.

There was then that quiet period between sticking dozens of applications in the mailbox and the first rejections; the one where your boss is happy because you have your focus back. By the time everything was done and dusted I had one university in the US wanting to take me down for an interview, and an offer of a post-doctoral position with David Reinhoudt in the Netherlands (plan B). Knowing now how departments in the US carry out faculty searches, I now appreciate that I had struck gold. I never saw the letters that John, Jim and Neville wrote, but they must have said some nice things. And so I went through the interview process at the University of New Orleans and did well enough to get an offer. I had my foot in the door! In a life-changing, bifurcated fork moment, I declined the post-doc in Europe with David, and Corrine and myself said farewell to New Your City and drove south. You don’t need a map to find New Orleans; you just follow the steep gradient in relative humidity until it reaches a maximum.

Like New Orleans itself, UNO Chemistry was a bit of a conundrum. Here was a relatively small university, recognized more locally for being a commuter campus than known nationally as a hive of research. But the Chemistry Department was an aberration on campus. It had a full complement of young, enthusiastic faculty, was receiving more federal research dollars than it had at any time in its history, and was publishing more high impact publications than ever before. Moreover, with 18–20 faculty and 60–70 graduate students there was a small but thriving research community; and one that was anticipating a move into a brand-new building.

We started setting up the lab in August 1996, the new building was not going to be ready for another 10 months, so the space we were given was, how to put this, an afterthought at the end of a long tiring meeting that occurred sometime before I arrived. Still, you could do chemistry in the space, and that’s exactly what we set about doing. I say we, because we’d arrived in New Orleans without securing a position for Corinne, and so as a prelude to seeking gainful employment she decided to help out setting up the lab and initiating some of the chemistry. And so before a student could join at the end of the semester, Corinne and myself had a small academic enterprise purring away in little more than a small, sweaty cupboard. It turned out that Corinne and myself worked very well together, and so slightly by accident this turned into a permanent working relationship. Corinne is the yin to my yang (and vice-versa), and our working relationship has taught me many things, including that the culture in the biosciences of having a permanent technician/lab manager(ess), as part of a research group is an excellent idea. Having a permanent pair of feet on the ground whilst you hover over the lab at 35,000 feet is good for everyone. Moreover, a permanent manageress helps ensure that the on-the-ground lab culture and history is maintained; an especially important point if you only have two or three graduate students in the group. I don’t think we’d have it any other way now, but since it is unusual in the US chemistry community to have a lab manageress – especially one your married to – it is a constant struggle to find funding.

**Scheme 5:**
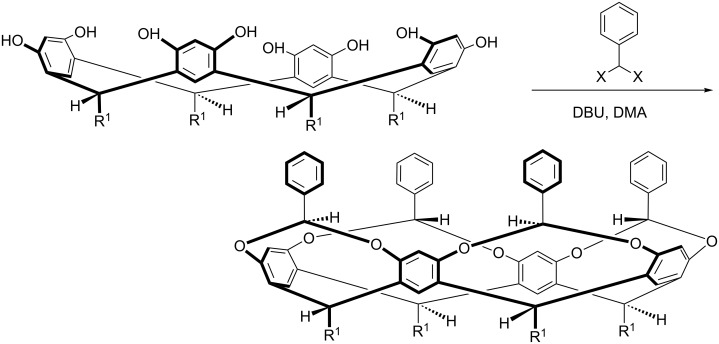
Stereoselective bridging of a resorcinarene with benzal halides.

Of the three proposals I’d written we decided to initially work on two: what was nicknamed the “chiral canyons project” and another project on the synthesis of deep-cavity cavitands. The first was inspired by Murray Goodman’s work [13] on the covalent templation of small, stable collagen-like triple-helices, and involved positioning a metal-ion coordinating template into a collagen structure such that the “active site” was situated in one of the chiral grooves of the triple-helix. In contrast, the second project on cavitands was focused on making a wholly synthetic hydrophobic pocket rather than one in a proteinaceous hybrid. We needed a peptide synthesizer for the canyons project and a round bottom flask for the cavitand work, and as we had to buy the former via a long, drawn-out purchasing process, and the latter could be picked up from the department store, the cavitand work was the first thing we did. The first new reaction carried out in our chemistry cupboard was to deepen the cavity of resorcinarenes (Scheme 5).

To attempt this stereoselective “bridging” of resorcinarenes we ordered both benzal chloride and benzal bromide; because both were in the Aldrich catalogue, and because, as I’m fond of telling students, you should never assume you know what Nature will do and should always maximize your chances when investigating a reaction. Anyway, both benzal halides were cheap (e.g., $35.15 for 25 g of benzal bromide; as the first compound we ordered I still have the (framed) order card on my wall). The idea was to quickly deepen the resorcinarene bowl, but the reaction involved making eight C–O bonds and creating four stereogenic centers in the shown, phenyl ring “up” configuration. If one ring pointed “in”, then the cavity was no more. I had my doubters with this reaction, and it failed miserably with benzal chloride (we fished out <5% of the desired cavitand). However, if short R groups such as methyl were avoided and benzal bromide was used instead, yields were near 60% in selected polar-aprotic solvents, i.e., ~93% yield per bond and ~88% diastereoselectivity [14–16]. Unfortunately a reviewer at the journal that we first submitted our manuscript to was also a doubter, even after we sent all the spectra, crystal structure information, and whatever other piece of data we thought might be useful to back up the text and supporting information. Alas, it was to no avail and the journal rejected the manuscript. It turned out that no information would have been sufficient, as the reviewer confided many years later, they just didn’t believe our yields. I guess that was kind of a compliment.

As well as being our first successful reaction in the lab, this benzal bridging was also a gateway to speaking at my first international meeting. I had no idea at the time, but one of the organizers of the meeting had unsuccessfully tried the same reaction a year or two earlier… using unfortunately benzal chloride. They never tried using the bromide, so when they saw my abstract they were naturally intrigued, and before long, I was invited to give a flash presentation at the meeting.

**Scheme 6:**
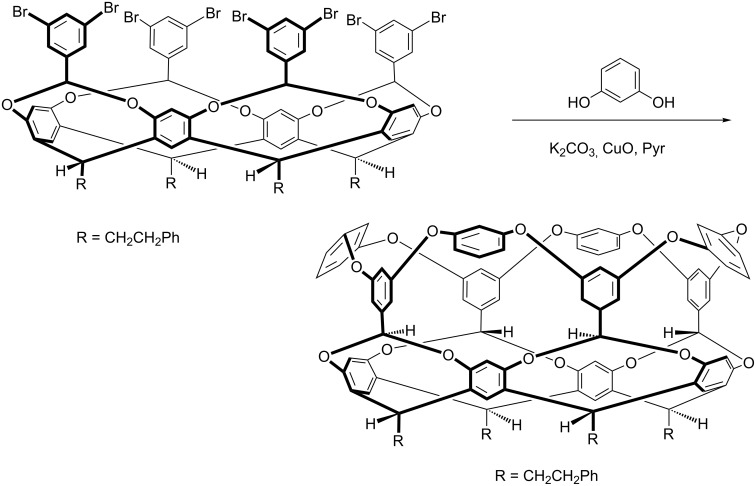
An eight-fold Ullman ether “weaving” reaction.

As we anticipated, the benzal-bridged cavitand shown in Scheme 5 was not a particularly good host; the free rotation of the aromatic rings ensures a poorly defined cavity. Moreover, although we could insert a range of functional groups into this second row of aromatic rims simply by using substituted benzal bromides [15–16], generally these cavitands were very acid sensitive. And so, using whatever means possible to avoid stray protons, we set out to rigidify the structure of the cavity. Many procedures were conceived, but the one most unlikely to succeed on paper – an eight-fold Ullmann ether reaction – was the one that ultimately worked best. Thus, treatment of the shown octa-bromide (Scheme 6) with resorcinol led to the insertion of a third row of aromatic rings and the corresponding deep-cavity cavitand [17–18]. This “weaving process” can be carried out with many resorcinol derivatives, but the necessity of using variations on the classical Ullman ether reaction (none of the milder modern-day variations have worked in our hands) does limit the product range somewhat.

These highly preorganized deep-cavity cavitands proved to be much better hosts, and the biggest determinant for guest binding beyond size compatibility with the pocket is the crown of four inward-pointing benzal C–H groups near the base of the pocket. Linked to two oxygen atoms, each C–H group is relatively electron deficient and therefore – at least on the scale of C–H hydrogen bonding – a reasonable donor. Synergistically however, four groups can readily control guest selection and orientation within the pocket. For example guests with halogen atoms bind relatively strongly by placing their halogen atom down into this crown [17–18]. Moreover, functional groups introduced into the entrance/rim of the host during the weaving step can also force guests to adopt specific orientations [19–21]. Beyond these structural features, the biggest determinant in guest binding was the solvent. Solvents such as DMSO gave the strongest complexations, whilst halogenated solvents gave the weakest. Apparently the former does not solvate the cavity well.

**Scheme 7:**
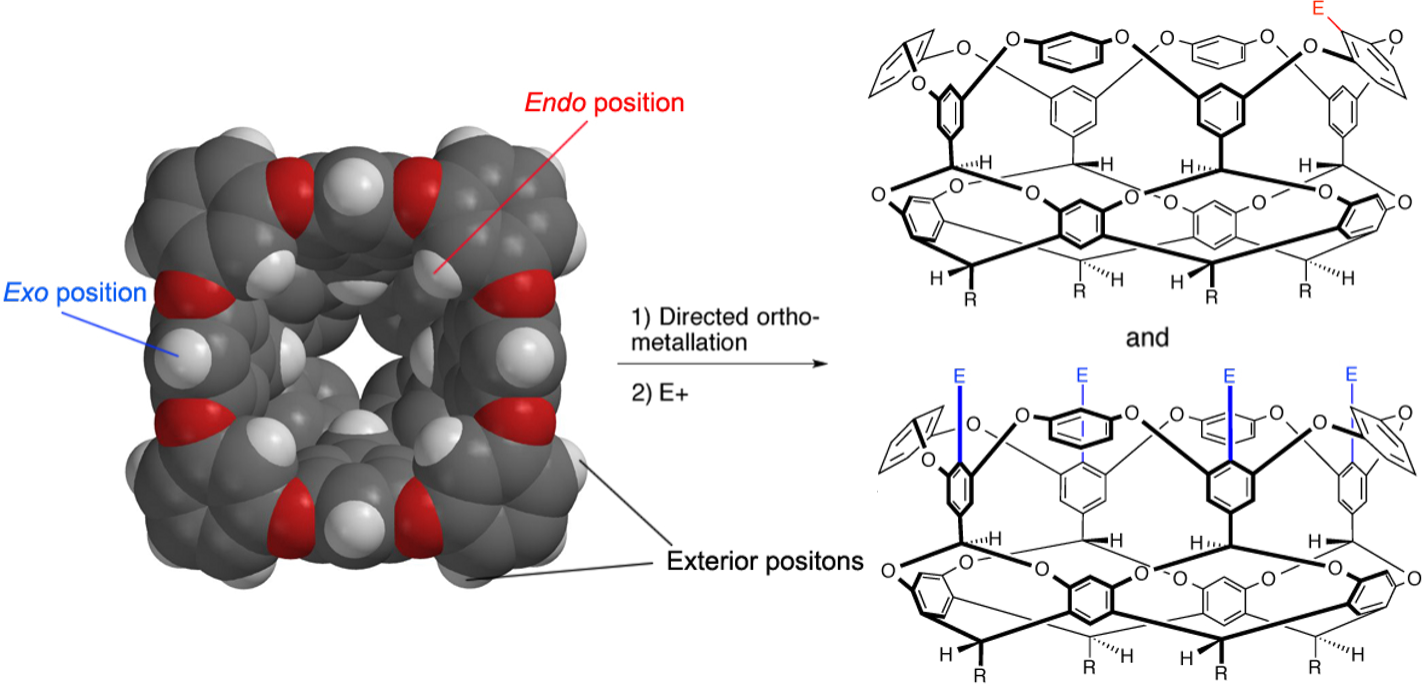
Directed *ortho*-metallation of the deep-cavity cavitands, showing the mono-*endo* substituted to tetra-*exo* substituted products.

One of the remarkable features of these cavitands is that although structurally quite complex, because of their well-defined structure they can be readily functionalized [22–23]. For example, directed *ortho*-metallation and quenching with electrophiles could at first blush theoretically lead to a plethora of products because there are sixteen aromatic carbons (labeled *endo*-, *exo*- and *exterior*- in Scheme 7) that are doubly activated by O-substituents around the rim of the pocket. It transpires that lithiation does not occur at the exterior positions of these hosts, so there are only (!) eight positions where reaction occurs. However, by control of stoichiometry and the nature of the organo-lithium only a grand total of ten different products of the possible sixty-nine can be formed. Hence by careful control of the conditions, compounds ranging from the mono-*endo* substituted to tetra-*exo*-substituted (Scheme 7) can be isolated in useful yields. The highly defined nature of the intermediate lithiate anions is important to the control of these reactions. Thus, much wider ranges of products are observed if direct electrophilic substitution is used as an alternative strategy of host functionalization [24].

**Scheme 8:**
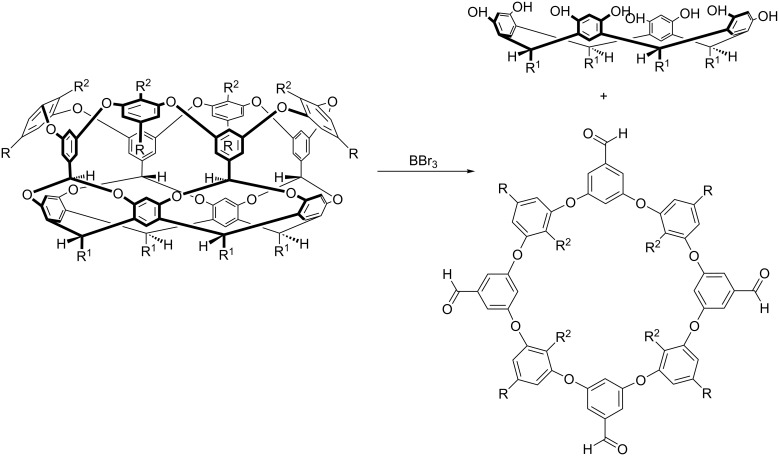
Macrocycle synthesis via resorcinarene covalent templates.

As well as proving to be useful hosts, these deep-cavity cavitands also demonstrated that resorcinarenes could be used as covalent templates to form macrocycles. For example, the deep-cavity cavitand shown in Scheme 8 can be treated with excess BBr_3_ to cleave the four, benzal-bridges and liberate a new family of aromatic macrocycle [25]. Interestingly, as these hosts are tetra-acetals we initially tried removing the template under acidic conditions. However, even reflux in 1:1 EtOH/H_2_SO_4_ for a week failed to affect the starting material. Evidently, under reversible conditions, a broken acetal can easily reform before the other three are themselves cleaved. An irreversible acetal cleavage mechanism is therefore key to success.

**Figure 4:**
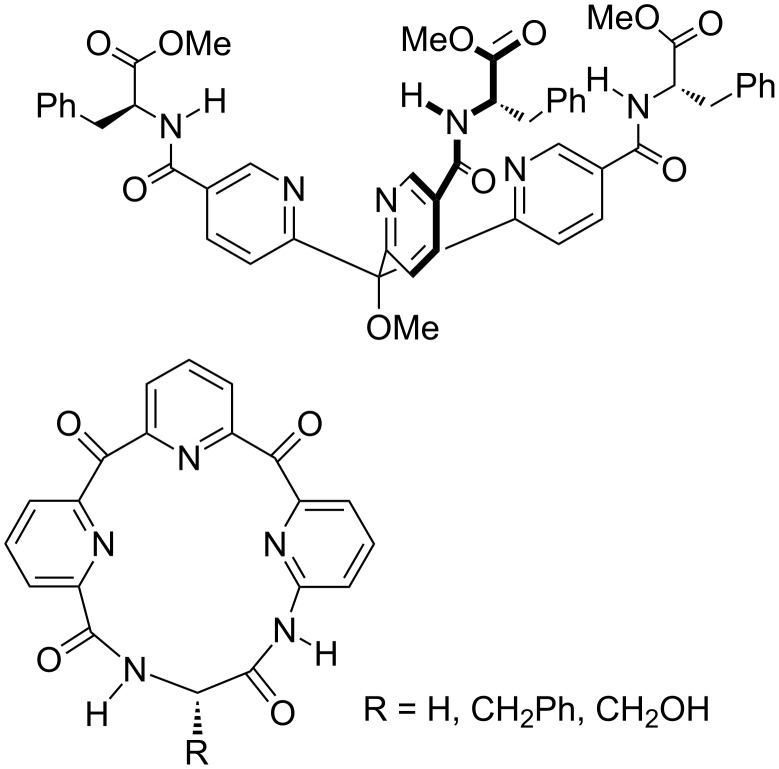
Tris-pyridyl hosts.

Meanwhile, because our chiral canyons project required some new, tripodal zinc-binding ligands for templating a collagen helix, the whole endeavor had itself become very syntheses oriented. We designed two general families of tris-pyridyl hosts to bind zinc ions within our proposed chiral canyons (Figure 4) [26–28]. The shown macrocycle turned out not to be a strong binder of zinc, but did demonstrate enantioselective binding of chiral amino acids that was dependent on the nature of the counter-anion [27–28]. On the other hand the shown tris-pyridylmethane ligand did bind zinc quite well, with its propensity to form the desired 1:1 host–zinc complex rather than a potentiometrically inert 2:1 complex controlled by the nature of the pyridyl substituents [26]. However, with the increasing momentum of the cavitand project it was proving harder to get students to work on this project, and with only a small group possible at UNO, taking these tris-pyridylmethane ligands into chiral canyons never materialized.

There was however a serious problem with our cavitand work: we were working in organic solvents, not water. Throughout my career, water had been just under the surface of all my research endeavors: I had been working towards water-soluble cavitands and making four-helix bundles that assembled via the hydrophobic effect at UBC, I had been working with water-soluble zinc complexes at NYU; even non-water-soluble steroids rely in part on the hydrophobic effect to bind. The common denominator was water, “the solvent of life”. However, although we understand water quite well, we are far from fully understanding aqueous solutions [29–30], and to be frank, it’s hard to contribute to this understanding if you are working in chloroform. Something had to be done.

**Figure 5:**
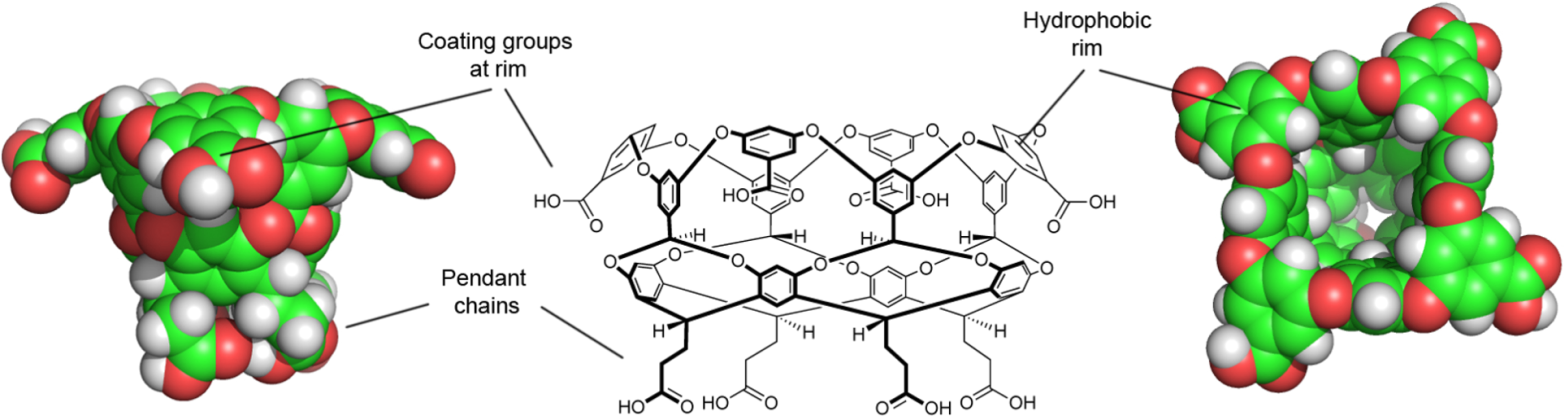
(Center) Chemical structure of the octa-acid host. (Left and right) Respective space-filling representations of octa-acid showing side and plan views.

We examined several approaches to making deep-cavity cavitands water-soluble, but the one that worked best led to what has been commonly called octa-acid (Figure 5) [31–32]. Now we had a host that: 1) was evenly “coated” with water-solubilizing carboxylic acids/carboxylates to bestow good water solubility under basic conditions, and; 2) possessed a rim of hydrophobicity around the entrance to its interior hydrophobic pocket. We envisioned that driven by the hydrophobic effect this host would readily form 1:1 complexes in water. More importantly, because the pocket and rim of the host would be poorly solvated, in the presence of an apolar guest we envisioned the host would dimerize around it. In other words, we hoped that the hydrophobic effect, would drive capsule assembly. To our knowledge this represented an entirely different approach to the assembly of container molecules; up until that point strategies such as hydrogen bonding or metal coordination were the focus of attention.

**Figure 6:**
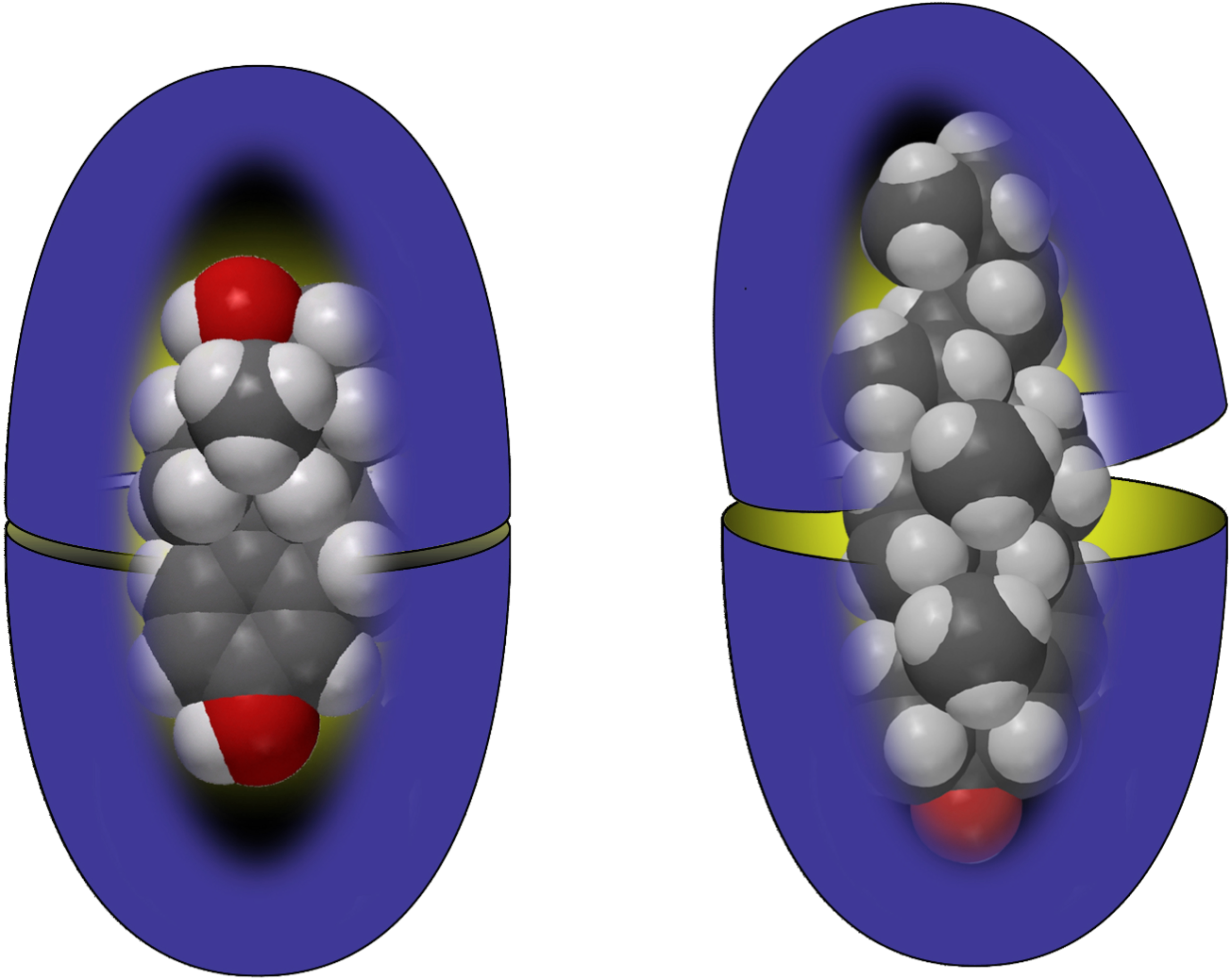
Cartoons of the 2:1 host–guest complexes of estradiol (left) and cholesterol (right).

Our first investigations with octa-acid looked at assembly around steroids [31]. There was obviously a personal reason for this, but more importantly we wanted guests that were very hydrophobic and rigid. Our concern was – and this proved to be completely unfounded – that two octa-acids in a capsule would slip and slide all over each other. In other words, with a lack of what are traditionally viewed as functional groups that can direct assemblies, the structural definition and kinetic stability of 2:1 host–guest complexes might be low. We therefore wanted to introduce a bias by using very rigid guests. We were therefore grateful to discover that a range of steroids, from as small as estradiol to as large as cholesterol formed exceedingly stable capsular complexes (Figure 6).

Regarding their stability, even at 5 μM, NMR showed complete encapsulation of estradiol; giving a minimum apparent binding constant for the empty capsule (the host is actually monomeric in the absence of guests) of 1 × 10^8^ M^−1^. When the steroid was very large, e.g., cholesterol, NMR showed that the capsular complex was near its carrying capacity, but the complex was still stable on the NMR timescale.

**Figure 7:**
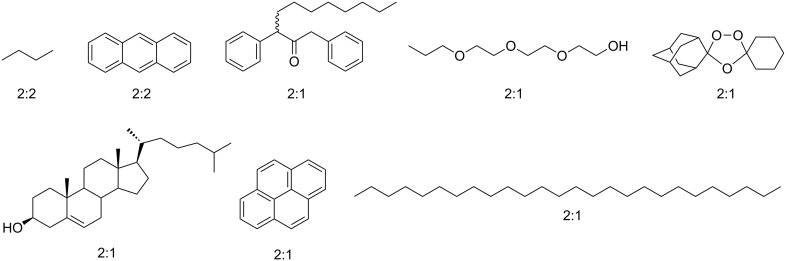
Representative guests for the capsular complexes formed by octa-acid (stoichiometry shown in parenthesis).

It transpires that any molecule that would energetically rather not be in water and is small enough to fit, forms a capsular complex with octa-acid. There are two caveats to this statement. First, very small guests such as methane form 1:1 complexes; the cut-off for simple alkanes is between ethane and propane; the former forms a 1:1 complex whilst the latter forms a kinetically stable 2:2 capsular complex. Second, if guest binding generates a rather hydrophilic portal region, then dimerization of the host will not occur. For example the binding of charged amphiphiles such as *n*-octanoate lead to 1:1 complexes [33–34]. With these two points noted, a wide range of guests trigger dimerization to form the capsule with ostensibly sixteen negative charges on its exterior and a dry, water-free nano-space for harboring a guest or guests. The range of guests that form 2:1(2) host–guest complexes is illustrated by the examples in Figure 7. As expected, if a co-solvent is added then these complexes are broken down. Thus, screening of eight different co-solvents for their ability to break capsular complexes revealed that the most powerful solvent for doing so is acetonitrile, whilst solvents such as DMSO and MeOH require three times as much to do likewise [35].

**Figure 8:**
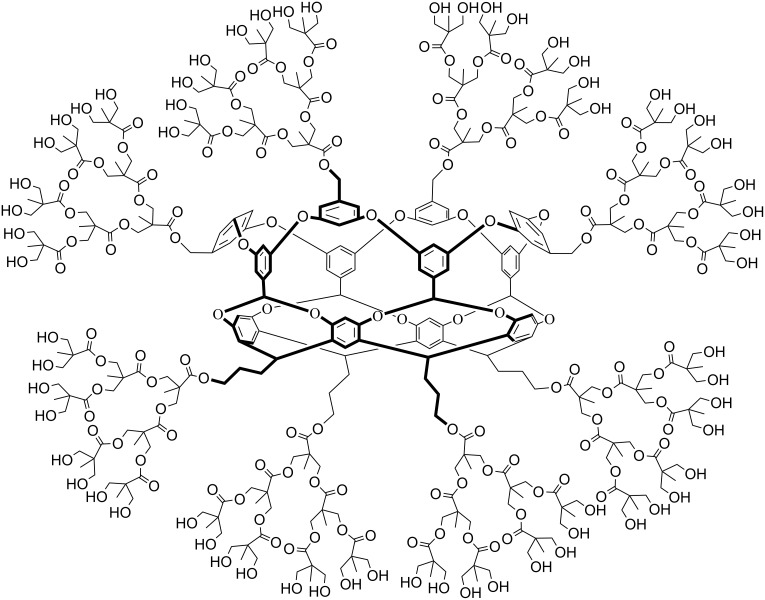
A dendrimer-coated cavitand.

Water solubility can be bestowed in many ways, and although eight negatively charged carboxylates have proven to be one of the most reliable approaches to date, it is not the only approach. For example, working with Scott Grayson across town at Tulane University we successfully formed the dendrimer–coated host shown in Figure 8 [36], as well as wide series of polymer coated derivatives coupled using “Click” chemistry [37].

**Figure 9:**
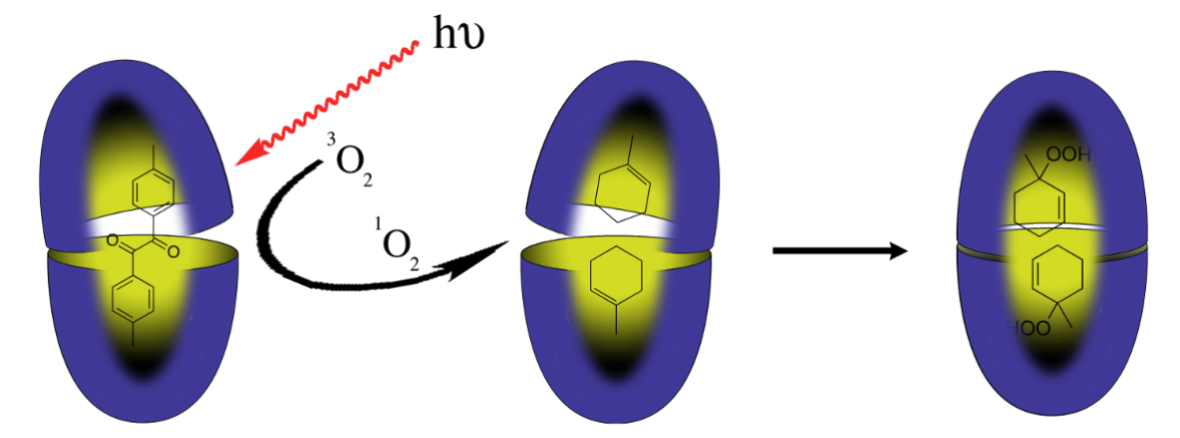
Selective oxidation of olefins by singlet oxygen.

Forming stable complexes for a broad range of guests, the capsules formed by octa-acid are ideal nano-scale (yocto-liter) reaction vessels. Our first foray into this topic relied on collaboration with Vaidhyanathan Ramamurthy, and examined a wide range of photophysics, and photochemical conversions [38–41]. One particularly enjoyable example of a reaction in the capsule formed by octa-acid came through collaboration between our group, Ramamurthy’s, and the group of the late Nicholas Turro (Figure 9) [42–43]. If a solution of a capsular complex of the sensitizer dimethylbenzil is irradiated, the encapsulated sensitizer is excited and can transfer its energy to an oxygen molecule adventitiously entering the capsule. (Although guest exchange is slow on the NMR timescale, there is a much faster dynamic breathing of the capsule that allows small molecule entry and egress [44].) The resulting singlet oxygen can escape the capsule, and if there is a second complex containing a substrate then the ^1^O_2_ can enter that cavity and react with the encapsulated substrate. In these experiments the substrate was a 1-methylcycloalkene, two copies of which bind to the capsule in specific, “methyl down” orientations. The first step of the reaction between singlet oxygen and the alkene is allylic hydrogen atom abstraction, and although there are three possible H-atoms for reaction, because of the specific guest-binding motif only an H-atom at the 3-position is removed. The result is therefore a 90% yield of the 1-peroxide rather than the 2- or 5-peroxide. In essence therefore the octa-acid not only facilitates reaction by dissolution in aqueous solution, it also engenders highly selective photochemical conversion via the transfer of chemical information – in the form of ^1^O_2_ – from one capsule to another.

**Figure 10:**
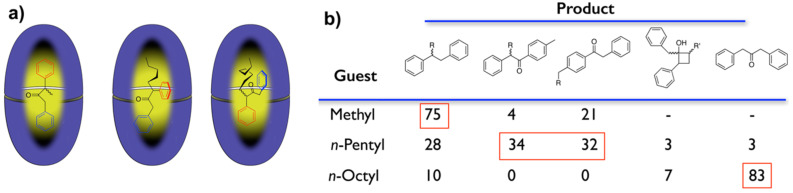
a) Preferred packing motifs of methyl, pentyl and octyl guests. b) Product distribution observed for the complexes of the same three guests.

A second interesting example of a reaction in the capsule formed by octa-acid illustrates the effect of guest packing upon reactivity [45]. Eight *n*-alkyl dibenzyl ketones (R = CH_3_ through *n*-C_8_H_17_) were found to form 2:1 capsular complexes capable of undergoing Norrish-type I and/or type II photoreactions. NMR analysis revealed that, depending on the size of the R group, the guests adopted one of three principle packing motifs (Figure 10a). In essence, the host acts as an external template to promote the formation of distinct guest conformations. As a result of their packing motif, the methyl, ethyl, and *n*-propyl dibenzyl ketones yielded large amounts of the decarbonylated product as well as sizable amounts of a rearranged product (Figure 10b). In contrast, the different packing motif of the *n*-butyl and *n*-pentyl guests yielded equimolar amounts of two rearrangement products. Finally, in further contrast the *n*-hexyl, *n*-heptyl, and *n*-octyl ketones yielded large amounts of Norrish-type II products. These results highlight the difference between macro-scale and nano-scale reactors: within yocto-liter vessels there are multiple opportunities for intimate contact between substrate and nano-reactor. The result is exceptionally high cage effects in which the two radicals that form within the capsule can only react with themselves. Moreover, these intimate contacts open up entirely new product landscapes by engendering rearrangement reactions not seen in solution.

**Figure 11:**
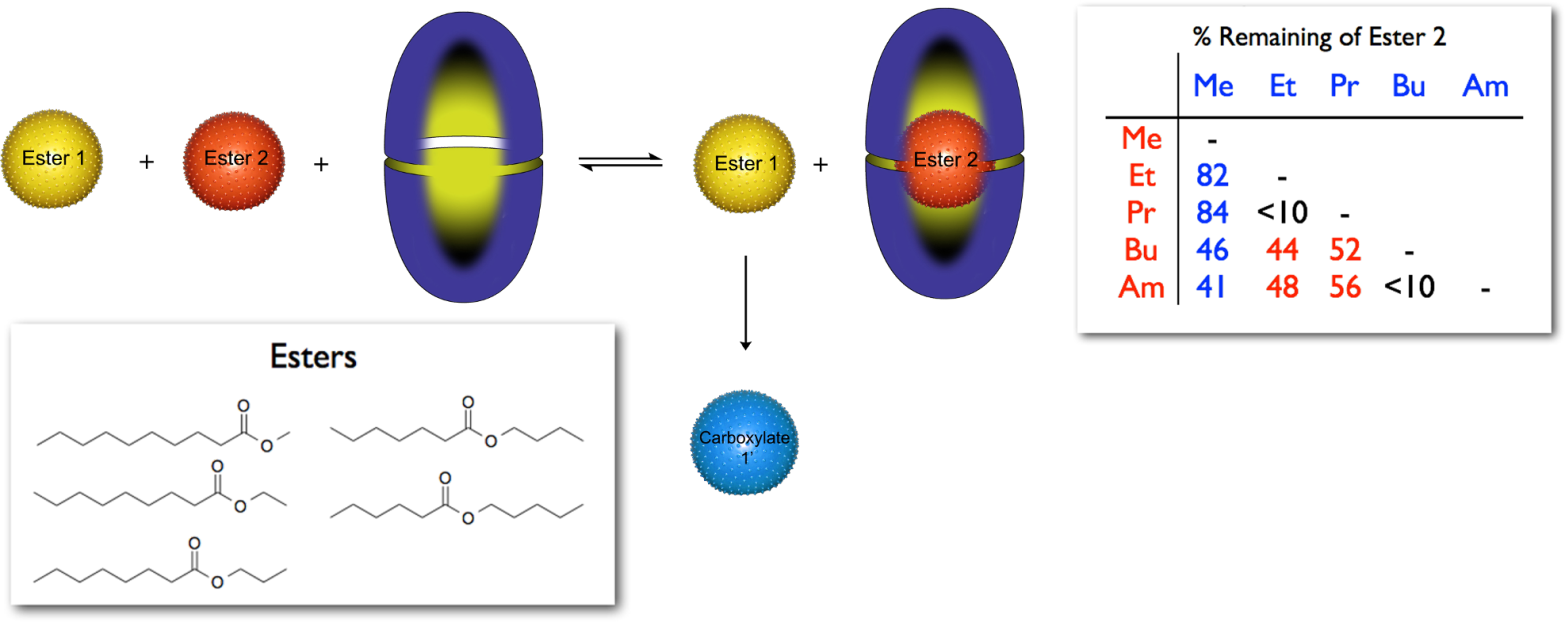
Schematic of the competition of two esters for the capsule formed by octa-acid. The ester that binds weakest to the host (arbitrarily Ester 1) remains in solution and is hydrolyzed to the corresponding carboxylate. The table shows the percentage of Ester 2 remaining after all of Ester 1 has been hydrolyzed. The color of the font of listed values indicates which ester of the pair predominated (red listed vertically, blue horizontally).

The flip-side of capsules as yocto-liter reaction flasks are capsules as a means of molecular protection, and whilst the former has continued to gain prominence, the latter was (and still is) poorly developed. To explore this idea we carried out the kinetic resolution of pairs of constitutionally isomeric esters in the presence of octa-acid [46]. The concept is straightforward. The chosen esters (Figure 11) are so similar that it is difficult to separate them chromatographically, and in basic solution they cannot be kinetically resolved because they undergo hydrolysis to the corresponding inseparable acids at approximately the same rate. How would the capsule formed by octa-acid affect this situation? To keep things manageable we focused on pairs of esters. In the presence of octa-acid one of the esters preferentially binds to the capsule and is kept “safe” in the confines of the container, whilst the weaker binding competitor is primarily left in solution and undergoes hydrolysis. Ideally therefore, none of the stored ester undergoes hydrolysis, before all of the second reacts. We discovered that the esters all formed kinetically stable 2:1 host–guest complexes and bound with the following relative affinity to the host: methyl > *n*-pentyl > *n*-butyl > ethyl > *n*-propyl. Furthermore, each of these complexes was exceptionally stable at room temperature (<5% reaction after 1 month). However, at 100 °C the hydrolysis rates were reasonable, and when pairs of esters were pitted in competition the kinetic resolutions shown in Figure 11 (table) were obtained. To account for this data we built a Michaelis–Menten model that showed how the kinetics of hydrolysis in the presence of octa-acid is dependent on both the equilibria between the free and bound species and the relative rate with which the free esters undergo reaction.

**Figure 12:**
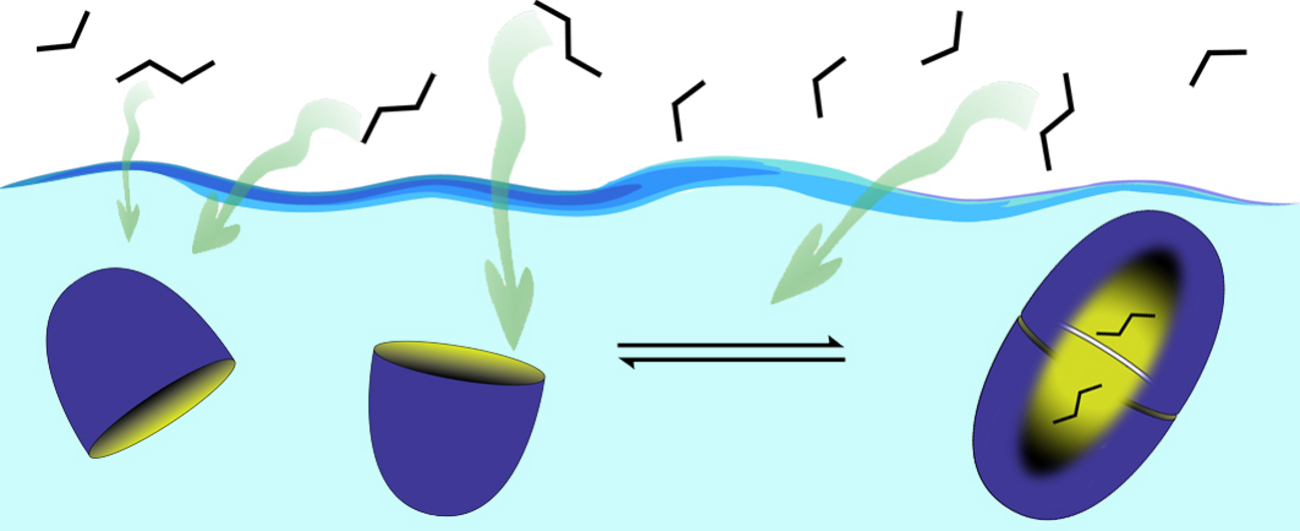
Schematic of the inter-phase separation of propane and butane; the latter binds more strongly to the capsule formed by octa-acid and so is selectively sequestered into aqueous solution.

The capsule formed by octa-acid is also capable of bringing about physical separations. One facile way to examine this is to consider the absorption of gases from the gas phase into a solution phase. We therefore examined the uptake of butane into an aqueous solution of octa-acid to form the corresponding 2:2 host–guest complex. In doing so we determined that it was possible to form methane solutions that were 200 times higher in concentration than the maximal solubility of the gas in water. The concentration of butane within the capsule itself was estimated to be ~0.5 M, i.e., akin to its supercritical state. If (for fun) this guest is treated as an ideal gas, then the pressure within the capsule can be calculated to be roughly 100 atmospheres. Other hydrocarbon gases could be encapsulated. For example, methane and ethane formed 1:1 host–guest complexes, and propane formed a 2:2 complex. Interestingly, the extra methylene group of butane meant that it bound to the host twelve times more strongly than propane, and this allowed selective butane complex formation from a 1:1 propane–butane mixture (Figure 12). In short, aqueous solutions can be used to separate gases; to paraphrase Cram, the synthesis of the host prepays the energy requirements for gas separation.

As discussed, the precise binding motif of an encapsulated guest – how it packs within the inner space of the capsule – is a significant factor in determining how guests undergo reaction. More generally, how a guest packs will dictate its overall affinity for the capsule and hence will affect any application that these types of complex are applied to. One straightforward approach to examining how flexible guests pack within these capsules is to consider the *n*-alkanes, the homologous series of which is available in pure form all the way up to C_50_H_102_. In considering this we have found that for the octa-acid dimer the boundary between two or one guest encapsulation is C_8_H_18_ [47], but that guests longer than C_12_H_26_ must compress in some manner in order to be accommodated. There are three principle ways that such large guests undergo compression [48]. For alkanes such as C_14_H_30_ the guest adopts a helical form; one that is of high energy in solution because of the gauche interactions down the length of the chain, but within the capsule maximizes non-covalent contacts between host and guest. With larger species such as C_18_H_38_ the guest must adopt a J- or U-shape within the cavity; a motif that is sometimes seen when fatty acids bind to fatty-acid transport proteins. Finally, the third and highest energy guest motif within the capsule can be observed with guests such as C_26_H_54_. This guest packs with each methyl terminus in separate poles of each hemisphere, but the bulk, central region of the guest forms a flattened disc that lies in the equator of the capsule prizing the two hemispheres apart. Thus in profile the bound guest resembles a spinning-top. This same study [48] also examined the consequences of covalently linking together two octa-acid molecules. For this “dimer” host, guests smaller than *n*-heptadecane (C_17_H_36_) bind to the host in a similar manner as they do to the assembled capsule of octa-acid. However, guests larger than C_18_H_38_ do not adopt a U-shaped packing inside a 1:1 complex, but instead induce the formation of a more capacious *D*_2_*_d_* dimeric assembly that allows the accommodation of two copies of the guest.

**Figure 13:**
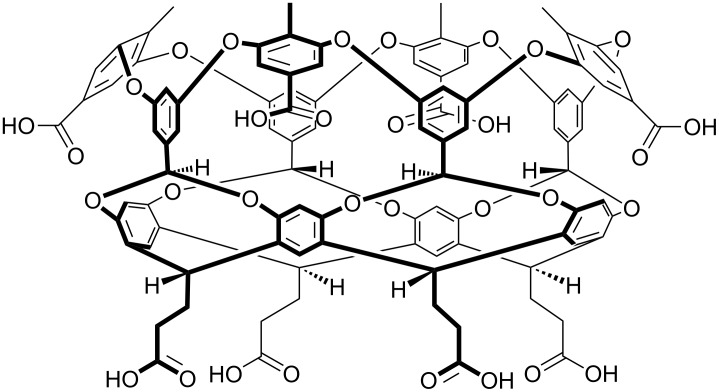
Structure of tetra-*endo*-methyl octa-acid (TEMOA).

A related deep-cavity cavitand that we have worked with is tetra-*endo*-methyl octa-acid (TEMOA, Figure 13). The four methyl groups at the rim of this host project inwards and upwards so as to subtly change the shape of the pocket by reducing the width of the portal but increasing its depth. The result is a host with approximately the same carrying capacity as octa-acid, but very different binding and assembly properties. For example, the binding profile of octa-acid using the metric of *n*-alkane complexation is as expected. The smallest of guests form 1:1 complexes, whereas with guests larger than ethane a 2:2 or 2:1 capsular complex is formed. In contrast the binding profile of TEMOA is non-monotonic: very small and medium sized guests form 1:1 complexes, whereas small and large guests form dimeric capsular assemblies [49]. This unique property comes about because the four methyl groups reduce the relative stability of the dimer capsule so that guests such as C_5_H_12_ promote the formation of a 2:2 capsule, but near the transition point from two to one internalized guest, C_7_H_16_ destabilizes the capsular form and leads instead to a 1:1 host–guest complex. Only with C_9_H_20_ – one copy of which can adequately fill the capsule – does the host revert to an assembled form. This unusual non-monotonic profile carries over when the formation of hetero-host complexes formed between octa-acid and TEMAO and *n*-alkane guests are examined [50].

**Figure 14:**
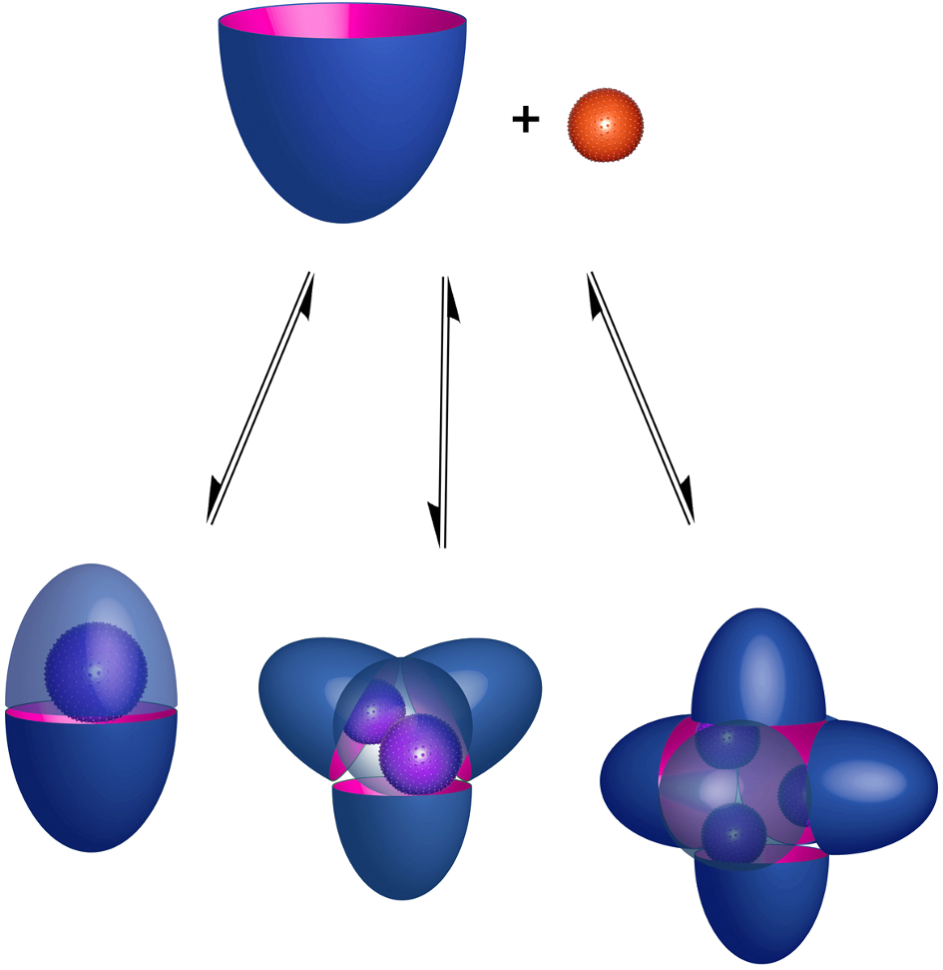
Assembly properties of TEMOA.

This reduced propensity of TEMOA to dimerize means that when the guest is as large as C_17_H_36_, rather than form a dimer the cavitand switches to a tetrameric, pseudo-tetrahedral, 4:2 host–guest complex (Figure 14) [51]. This is possible because the methyl groups that sterically hinder dimerization interfere less with an assembly as the angle between the two interfacing surfaces of a pair of cavitands increases from 0° (in a dimer) to ~70.5° (in a regular tetrahedron). This senary assembly many be higher in free energy, but because this container has a volume of four hosts *plus* that of a roughly tetrahedral volume in the center of the assembly, the guests are afforded more space: ~1400 Å^3^ in total. The assembly properties of TEMOA do not end there. With even larger guests such as C_24_H_50_ the host forms the corresponding octahedral 6:3 nonary complex with an internal volume of ~3400 Å^3^, i.e., the volume of the six cavitands plus the roughly cubic central void at the center of the inner space. This represents one of the largest container systems synthesized to date, and certainly the largest synthesized with the help of the hydrophobic effect.

To summarize, the combination of octa-acid, TEMOA and the hydrophobic effect have allowed us to form mono-dispersed 1:1, 2:1, 2:2, 4:2 and 6:3 complexes with molecule weights up to ~11.5 kD. Furthermore, these entities demonstrate that orchestration of the hydrophobic effect can be used to bring about a range of unique phenomena, including novel chemical conversions, and chemical or physical separations.

Concomitant to using the Hydrophobic effect to engender unusual phenomena such as those outlined above, we have also been involved with improving Science’s understanding of both the Hydrophobic effect and the Hofmeister effect [29]. Contributing to understanding these phenomena is truly an interdisciplinary affair, and in particular, over the last few years we have collaborated with computational chemists whose unique skills allow us to “visualize” what water is doing within the solvation shell of solutes. One exciting facet of these collaborations is the SAMPL exercise in which we and other supramolecular chemists a priori determine the thermodynamics of complexation for a series of processes, and keep the results under wraps whilst computational groups around the world attempt to predict the data [52]. This competition gives the computationalists a unique opportunity to try and hone their skills, which in return provides feedback to those interested in the a priori prediction of the thermodynamics of binding. For example, being able to predict ahead of time which ligands binds best to a protein binding-site has the potential to save the pharmaceutical industry billions of dollars in drug development.

Collaboration with my former colleague Steven Rick has provided considerable insight into the solvation of octa-acid. Briefly, this work showed that between 0–7 (~4.5 on average) water molecules can be found within the hydrophobic pocket [53]. These water molecules are relatively high in energy, in so much as they cannot form their regular complement of hydrogen bonds seen in the bulk (~3.6) but instead must form weaker interactions with the concavity; and the deeper the water molecule the higher its energy and the lower its translational diffusion constant, but the higher its rotational diffusion constant. Overall however, the pocket would rather be solvated: Δ*G*_hyd_ Δ*H*_hyd_ and *T*Δ*S*_hyd_ were determined to be approximately, –5, –20, and 15 kcal mol^−1^ for 4 or 5 water molecules.

The addition of salts to water or aqueous solutions lead to a plethora of phenomenon all classified under the banner of “the Hofmeister effect”. The classical manifestation of the Hofmeister effect is the ability to increase or decrease the apparent solubility of organic solutes dissolved in water. Thus for example, sodium sulfate will decrease the solubility of a protein causing it to precipitate from solution. In contrast, salts such as sodium thiocyanate cause proteins to become more soluble, and in doing so break apart their tertiary structure. For a long time it has been appreciated that it is the anion that plays the more significant role in this Hofmeister effect; the question is how [54]?

We first started to investigate the Hofmeister effect in 2005 when we were on a forced group sabbatical at the University of Texas, Austin. The principle driving force for our near year long relocation was hurricane Katrina that devastated New Orleans, south east Louisiana, and southern Mississippi in the fall of 2005. One of my best memories of this difficult period arose from the twelve offers we received from colleagues around the country (and indeed beyond!) to temporarily move and set up shop in University X. We chose UT Austin, both because it was possible to get to New Orleans with an eight hours drive (important for rebuilding our house and helping rebuild the department), and because of the Department’s supramolecular strengths. Indeed, the coordinated and gracious charity of Jonathan Sessler (provider of research funds whilst we sought emergency support from the likes of the National Science Foundation and Department of Energy) and Eric Anslyn (who provided us with research space) was too attractive to turn down; especially since Jonathan’s neighbors, Syd & Arnie Popinsky, were offering us a room for the first week or two until we settled in.

Although a much larger department than UNO, UT Austin had a poorer ratio of NMR instruments to students, and so we focused mostly on isothermal titration calorimetry (ITC) based research. There was the small matter of sneaking into a closed city and slipping under the radar of the National Guard to “steal” our own hosts, reagents, and ITC instruments; but that story is perhaps for another time. Back in Austin with everything we needed, we got down to some research… and of course filling out countless forms for insurance claims and requests for financial aid from the Federal Emergency Management Administration (FEMA).

Our first results that came out from this period demonstrated that the thermodynamics of guest binding to octa-acid were extremely perturbed by the presence of different salts [55]. As expected, the Hofmeister effect was indeed in operation: salting-out sodium sulfate increased guest association, whilst salting-in sodium perchlorate weakened binding considerably. The surprise was that the root cause of the latter was competitive binding of the anions to the hydrophobic pocket of our anionic host. A follow-on full paper expanded in these results considerably and provided a detailed thermodynamic picture of how the concentration and nature of salts influence hydrophobic solute association [56].

But why did some salts cause binding to increase? We answered this question back in New Orleans, not at UNO, but rather at Tulane University. Being a private institution Tulane had weather Katrina relatively well. Moreover, it had also weathered the withering Louisiana politics of the last decade that has resulted in the largest cuts to higher education in the nation. One metric of the effects of this political and economic upheaval was that between Katrina and the present day, the UNO chemistry faculty decreased from 18–20 to 5; with a concomitant decrease in the graduate student and post-doc population. Sometime before the Department numbers bottomed-out, the opportunity to move across town to Tulane arose and I accepted. It was a hard decision leaving UNO in such a state; things had been going so well before the storm. But if you think doing science is hard, try doing it in a dispiriting environment where no institutional decision was ever favorable (or even neutral). To take but one example, despite the best efforts of our then Chair (Matt Tarr), when I left the department it had functioned for two years with no secretary whatsoever.

**Figure 15:**
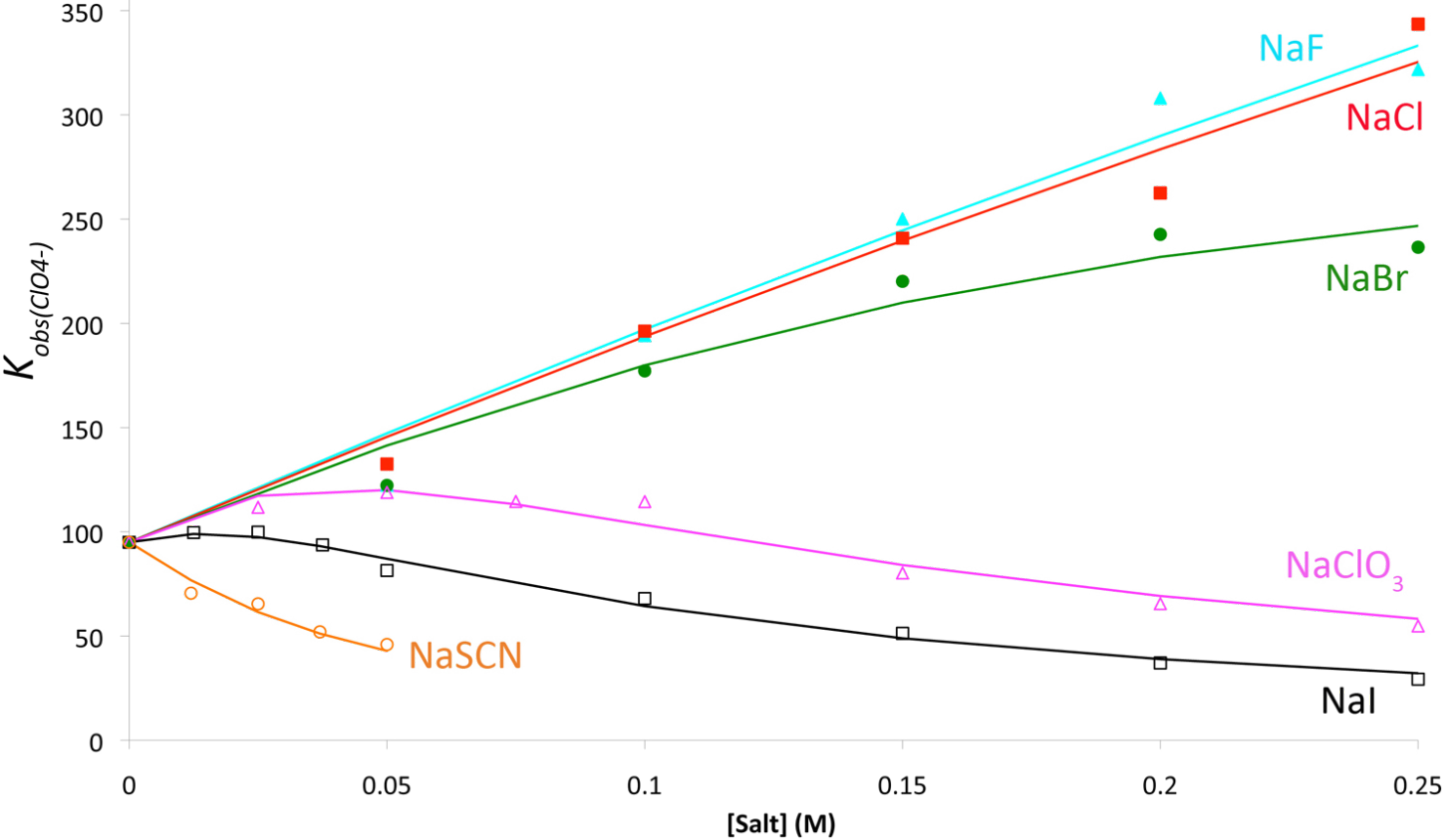
How salts influence the association constant (*K*_a_) for the binding of ClO_4_^–^ to octa-acid (Figure 4). The indicated points correspond to NMR-derived data, whilst the lines correspond to the model devised (see text).

Back to the science. Why did some salts cause guest binding to strengthen? An answer came when we studied the binding of a much weaker guest to octa-acid; namely perchlorate [57]. Just as we observed with the binding of hydrophobic guests, perchlorate association was influenced by the presence of other salts: sodium chloride and fluoride caused the affinity to increase whereas sodium thiocyanate caused its affinity to decrease (Figure 15). The data from this NMR study allowed us to build a model for how *K*_a_ changed as a function of salt concentration. Briefly, two factors accounted for >90% of the observed changes in perchlorate affinity as a function of co-salt ionic strength: where present, anion binding to the hydrophobic pocket caused perchlorate affinity to decrease, but in all cases sodium ion condensation to the exterior carboxylates of the host reduced its negative charge and hence increased anion affinity.

Cation binding to groups such as carboxylates is well documented, but why would an anion bind to a hydrophobic pocket? Elsewhere it has relatively recently been demonstrated that such salting-in anions have an affinity for the air–water interface, and furthermore it has been suspected for some time that the same anions have a weak affinity for the macromolecule–water interface (see references in references [29] and [54]), at least in part because such ions have relatively low free energies of hydration. Still, these energies are high, and it is frequently commented on in supramolecular chemistry how difficult ion recognition in water is precisely because this solvation shell has to be removed. But does it? Our latest Hofmeister-related paper revealed detailed thermodynamic data for a wide range of anions binding to octa-acid, and suggests that complexation only involves partial ion-desolvation [58]. These results builds on our earlier in silico studies [53]; anion binding to the pocket appears to involve simply replacing one or two high-energy water molecules with the anion. The details of why this should be are yet to be determined.

## Conclusion

So that’s roughly where we are regarding our exploration of the hydrophobic effect, the Hofmeister effect, and what can be done with them. Our research over the last two decades or so has revealed that it is possible to harness the properties of water to engender molecular containment and a range of new phenomena. More importantly perhaps, in trying to understand why we see what we see we are revealing new information about the hydrophobic and Hofmeister effects that dovetails with many aspects of recent water research carried out within other fields. Gratifyingly however, there is still a lot to learn about the interactions between water, salt and solute. Here’s hoping the next two decades will be at least equally revealing!
